# Large Granular Lymphocytic Leukemia: From Immunopathogenesis to Treatment of Refractory Disease

**DOI:** 10.3390/cancers13174418

**Published:** 2021-09-01

**Authors:** Misam Zawit, Waled Bahaj, Carmelo Gurnari, Jaroslaw Maciejewski

**Affiliations:** 1 Taussig Cancer Center, Cleveland Clinic, Translational Hematology and Oncology Research Department, Cleveland, OH 44106, USA; zawitmm@ucmail.uc.edu (M.Z.); BAHAJW@ccf.org (W.B.); carmelogurnari31@gmail.com (C.G.); 2 Division of Hematology and Medical Oncology, University of Cincinnati Medical Center, Cincinnati, OH 45229, USA; 3 Department of Biomedicine and Prevention, PhD in Immunology, Molecular Medicine and Applied Biotechnology University of Rome Tor Vergata, 00133 Rome, Italy

**Keywords:** large granular lymphocytic leukemia, refractory disease, immunogenomics

## Abstract

**Simple Summary:**

Large Granular Lymphocytic Leukemia (LGLL) is a clonal disorder of cytotoxic T-cells. Because of the variety of clinical presentations ranging from the mere presence of lymphocytosis to cytopenias and autoimmune conditions, this rare lymphoma may require treatment to control such manifestations. Although first-line treatments are more established, refractory cases are often managed based on the experience of the attending physician. Herein, we review the pathways involved in the pathogenesis of LGLL, including refractory cases, inferring clues as to the potentially actionable targets.

**Abstract:**

Large Granular Lymphocyte Leukemia (LGLL) is a rare, chronic lymphoproliferative disorder of effector cytotoxic T-cells, and less frequently, natural killer (NK) cells. The disease is characterized by an indolent and often asymptomatic course. However, in roughly 50% of cases, treatment is required due to severe transfusion-dependent anemia, severe neutropenia, or moderate neutropenia with associated recurrent infections. LGLL represents an interesting disease process at the intersection of a physiological immune response, autoimmune disorder, and malignant (clonal) proliferation, resulting from the aberrant activation of cellular pathways promoting survival, proliferation, and evasion of apoptotic signaling. LGLL treatment primarily consists of immunosuppressive agents (methotrexate, cyclosporine, and cyclophosphamide), with a cumulative response rate of about 60% based on longitudinal expertise and retrospective studies. However, refractory cases can result in clinical scenarios characterized by transfusion-dependent anemia and severe neutropenia, which warrant further exploration of other potential targeted treatment modalities. Here, we summarize the current understanding of the immune-genomic profiles of LGLL, its pathogenesis, and current treatment options, and discuss potential novel therapeutic agents, particularly for refractory disease.

## 1. Introduction to Large Granular Lymphocytic Leukemia

Large Granular Lymphocytic Leukemia (LGLL) represents about 2–5% of chronic lymphoproliferative disorders and predominantly affects the elderly (median age at diagnosis 66 years) [1]. Although the reported incidence is around 0.2 cases/1,000,000, the true prevalence may be much higher due to a large proportion of indolent, undiagnosed cases and, often, an inability to distinguish it from reactive cytotoxic T-cells (CTL) lymphoproliferation. The majority of LGLL cases are of the T subtype (i.e., derived from CTL), whereas only <10% are of natural killer (NK) cells origin [2].

LGLL is a heterogeneous and complex disease, and the etiology is not fully understood. In addition to genetic mutations (see below), some reports suggested possible viral infections acting as triggers for exuberant CTL responses. These included Epstein–Barr virus (EBV), hepatitis B and C, human T lymphotropic virus (HTLV), and human immunodeficiency virus (HIV) [3,4,5]. The chronic exposure to uncleared viral antigens may generate oligoclonal to clonal CD8+ cells expansions, and the occurrence of isolated, canonical *STAT3* mutations points toward neoplastic transformation of an originally reactive process [6].

T-LGLL includes both CD8^+^ and CD4^+^ (either alone or in association with CD8^dim^) subsets. In addition, according to the T cell receptor (TCR) rearrangement, the disorder can be further subdivided into T-LGLL αβ^+^ and T-LGLL γδ^+^ variants. The majority of cases represent an activated post-thymic cell proliferation expressing CD3^+^, CD4^−^, CD5^dim^, CD8^+^, CD16^+^, and CD57^+^, mostly with TCR αβ^+^ restriction. Of note, CD95 (Fas) is over-expressed on LGLL cells along with elevated intracellular and serum CD95L (Fas-L), suggesting intrinsic resistance to Fas-mediated apoptosis [2,7]. Recurrent gain-of-function *STAT3* mutations can be found in up to 40% of patients, whereas *STAT5* lesions are identified much less commonly and with preferential presence in specific disease subtypes (see Section 2.1) [8,9,10]. Of note, these mutations characterize LGLL with different disease features and clinical impact [11].

The diagnosis of LGLL is based on evidence of chronic clonal T- or NK-cell proliferation via cytology, immunophenotypic analysis, and TCR repertoires assessment. In cytology, circulating LGLL cells should be ≥0.5 × 10^9^/L in the peripheral blood smear [12]. Cell phenotype by flow cytometry helps in identifying T-cells and NK-cells with an aberrant phenotype. In particular, T-LGLL shows CD3^+^, CD8^+^, and CD57^+^ expression whereas NK-LGLL expresses CD8^+^, CD16^+^, and CD56^+^ [2]. The evidence of T-LGLL clonal expansion may be assessed by using TCR *γ*-polymerase chain reaction analysis and Vβ gene rearrangement testing, which help to distinguish between reactive and leukemic LGLL cells. In rare situations, a bone marrow biopsy with immunohistochemistry studies may be helpful, especially when LGLL counts are too low or in the case of the NK-subtype because, in this case, it is not possible to study TCR-repertoires’ clonality [12,13].

**Figure 1:**
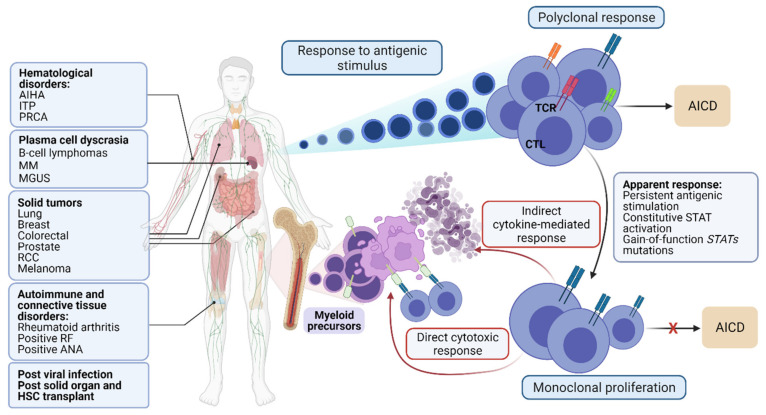
Diseases commonly associated with LGLL and schematic representation of underlying pathogenesis. MGUS: Monoclonal gammopathy of undetermined significance; PRCA: pure red cell aplasia; AIHA: autoimmune hemolytic anemia; MDS: Myelodysplastic syndrome; HSC: hematopoietic stem cell. AICD: activation-induced cell death; STAT: Signal Transducer and Activator of Transcription 3; TCR: T-cell receptor; CTL: cytotoxic T-cells; ITP: Immune thrombocytopenic purpura; MM: multiple myeloma; RCC: Renal cell carcinoma; RF: rheumatoid factor; ANA: antinuclear antibodies. Figures were generated with BioRender.com (accessed on 20 August 2021).

Unlike other lymphomas, LGLL expansion is usually self-limited, persistent, and not associated with lymphadenopathy or “B symptoms”, although splenomegaly is present in about 24–50% of cases [14]. Cytopenias, a major presentation of LGLL, may result from direct cell-mediated targeting of myeloid precursors (neutropenia) or erythroid precursors (reticulocytopenic anemia), or indirectly through cytokine-mediated destruction of hematopoietic stem cells, representing an indication for treatment [15]. In addition to cytopenia(s), LGLL is frequently associated with autoimmune disorders or other accompanying conditions (Figure 1) [16]. The pathogenetic link to these frequently co-occurring conditions remains speculative.

Clinically, LGLL patients may remain asymptomatic for many years due to the aforementioned indolent course of the disease [1,17]. However, severe neutropenia, moderate symptomatic neutropenia, transfusion-dependent anemia, or autoimmune hemolytic anemia may necessitate therapy.

## 2. Overview of the Main Pathways Involved in the Pathogenesis of LGLL with Possible Therapeutic Implications

The pathogenesis of LGLL suggests potential new therapeutic approaches, many of which can be adopted from the therapeutic armamentarium designed for autoimmune diseases. Indeed, multiple pathways also involved in immune responses are found to be dysregulated in LGLL. Two major categories can be identified: (i) pathways promoting survival and (ii) pathways contributing to cell ability to escape apoptosis [2,18].

### 2.1. Survival Promoting Pathways

The JAK-STAT signaling pathway is essential for the transduction of proliferative signals from the IL-6 receptor. Canonical *STAT3* variants (*Y640F, D661V, D661Y, N647I*) have been identified in 40% of LGLL patients, whereas *STAT5b* variants (*Y665F, N642H*) are prevalent in specific disease subtypes as they were recently reported in up to 19% of TCRγδ LGLL and up to 55% of CD4^+^ LGLL cases [10,19,20]. The finding of constitutive activation of JAK/STAT signaling and the frequency of *STATs* mutations point towards a pivotal role of this pathway in LGLL pathogenesis, possibly indicating scenarios for the treatment of this disorder with JAK inhibitors [1,9,21,22,23]. The JAK/STAT pathway is involved in a multitude of molecular functions, including transduction of signals conveyed by cytokine receptors such as growth hormone, erythropoietin, and interleukin-6 receptors [24]. Following dimerization, *STAT* translocates into the nucleolus and exerts its functions by activating gene expression (i.e., the pro-survival genes *BCL-2* or *MCL-1*), ultimately promoting LGLL cells survival by generating an auto-amplification loop involving autocrine and paracrine IL-6 and IL-17 production [12,24].

The Nuclear Factor kappa β (NF-κβ) signaling pathway is another transcription factor that has been implicated in the constitutive activation of LGLL cells. Once NF-κβ protein is translocated to the nucleus, it activates several proto-oncogenes such as *cyclin D1*, *c-myc*, and the anti-apoptotic genes *BCL-2* and *MCL-1*, promoting the production of IL-2 [23,25] and allowing LGLL cells to escape the physiological mechanism of activation-induced cell death (AICD) [23,26] (Figure 1).

Platelet-Derived Growth Factor (PDGF) receptor heterodimeric complexes (aa, bb, and ab) can be found on CTLs. An increased expression of both PDGF receptor type b and PDGF-bb results in an autocrine loop and activation of several pathways involved in LGLL (e.g., JAK-STAT, Ras-RAF-MEK1-ERK, and PI3K-Akt) [23,27]. Consistent with these findings, antibodies targeting PDGF-bb were shown to be cytotoxic to LGLL cells [28]. Whether activated by PDGF pathways or other mechanisms (see also below), RAS-RAF-MEK1-ERK signaling is hyperactive in LGLL (particularly in the NK subtype), and its inhibition showed encouraging results in this setting via restoration of Fas sensitivity [23,29]. It is worth mentioning that a significant shift towards pro-survival sphingolipids such as sphingosine-1-phosphate (S1P) has been identified in LGLL along with downregulation of the corresponding proapoptotic ceramide and sphingosine [30]. In particular, the upregulation of rate-limiting enzymes in LGLL such as sphingosine kinase 1 (SphK1), converting sphingosine to S1P, and the upregulation of N-acylsphingosine amidohydrolase 1 (ASAH1), converting ceramide to sphingosine, may represent one of the mechanisms responsible for enhanced LGLL cell survival [23].

### 2.2. Evasion of Apoptosis Pathways

The Fas/Fas-L pathway is essential in AICD [26]. Once Fas is activated by Fas-L, a cascade of molecular interactions in the cytoplasm of activated lymphocytes ensues, resulting in the formation of the death-inducing signaling complex (DISC), consistent with the Fas receptor, Fas-associated death domain (FADD) protein, and pro-caspase-8, ultimately leading to cell apoptosis [26]. Fas receptor, Fas-L, and soluble Fas (sFas) have all been found to be overexpressed in LGLL. In this process, sFas counterbalances FasL, resulting in the deactivation of the Fas/FasL pathway [7,31], a mechanism that may be deregulated in LGLL.

IL-15 is a member of the IL-2 family that controls the activation and proliferation of T- and NK-cells. This cytokine is produced by antigen-presenting cells (APCs) and exerts its effects through interaction with the soluble or membrane receptor IL-15Rα. This receptor presents IL-15 to IL-2/IL-15Rβ subunits, which are expressed in both T- and NK-LGL cells. In particular, IL-15 forms a complex with IL-2Rβ, finally resulting in activation of JAK-STAT and Ras/MAPK pathways, in addition to an unbalanced generation of antiapoptotic (Bcl-2 and Bcl-xL) and pro-apoptotic signals (Bim and Puma) [32]. Furthermore, it has been shown that LGLL cells overexpress CD122 (a receptor subunit shared by IL-2 and IL-15). Increased soluble IL-15Rα concentration has been found in patients’ sera, pointing towards an essential role of the IL-15 signaling pathway in LGLL pathogenesis and, possibly, treatment [27,32,33,34].

The PI3K-AKT signaling pathway may also be involved in the exuberant clonal expansion of CTLs. When activated, e.g., by the RAS-MAPK cascade [35], it leads to phosphorylation of PIP2 to generate PIP3, phosphorylating AKT and, consequently, mTOR. The proinflammatory proteins responsible for activation of this pathway have been found to be upregulated in LGLL patients [36], as shown by the higher levels of phosphorylated AKT in T-LGLL cells [37]. Furthermore, AKT blocks the inhibition of NF-κβ and perturbates the equilibrium of Bcl-2 and procaspase-9 [38]. Consistent with this role, PI3K inhibition induced apoptosis and decreased ERK1/2 expression in an in vitro T-LGLL model [37].

## 3. Current Therapy Approaches and Their Results

Due to the lack of randomized prospective trials, current standard treatment options in LGLL mostly rely on the metanalysis of phase II trials and case series. The mainstay of first-line therapies involves immunosuppression administered in a chronic and protracted fashion, rather than in pulse/cycle mode as is typical in many B cell lymphomas. This route of administration (chronic vs. pulses) is preferred because the proliferative fraction of memory cells for LGLL clone is low. The greatest amount of experience has been reported with methotrexate (MTX, 10 mg/m^2^/weekly), cyclosporine A (CsA, 3–5 mg/kg/day), or low dose cyclophosphamide (Cy, 50 to 100 mg/day), with or without short prednisone taper (Table 1). Treating patients for at least 4–6 months is recommended before assessing response [1,2].

In addition to its effects on DNA replication, MTX suppresses the activation of JAK/STAT signaling [39]. Indeed, *STAT3^Y640F^* mutant cases appear to be more likely to respond to MTX treatment [40], which is also the preferred choice when treating LGLL patients with associated rheumatoid arthritis (RA), neutropenia, or other autoimmune conditions [17]. CsA, as a calcineurin inhibitor, blocks NF-AT, IL-2, and IFN-γ expression [26]. Interestingly, the HLA-DR4 allele has been found to be overrepresented in LGLL patients with co-occurring RA. Moreover, it has been shown that HLA-DR4 carriers may have a higher likelihood of CSA responsiveness, suggesting the presence of underlying antigen-driven mechanisms in different contexts characterized by specific immunogenetic predisposition [41]. Cy appears to be a good option, especially for cases with concurrent PRCA or profound anemia [17]. Chronic daily oral administration seems to be the preferred approach rather than periodic intravenous (IV) boluses. However, it is worth noting that a lack of response to either MTX, CsA, or Cy is not uncommon, and a switch between the three should be considered in such cases [2]. In addition, Cy should not be used for more than 12 months to avoid long-term complications, as demonstrated by studies involving large cohorts of patients with rheumatologic conditions [2]. Refractory cases are not uncommon and usually present with persistent transfusion-dependent anemia or severe neutropenia, requiring >1 line of treatment in up to 30% of cases [42,43]. Response to therapy (Table 1) varies between cohorts, and patients often require trials, switching among different treatments.

## 4. Salvage Second-Line Therapeutic Approaches

A variety of immunosuppressive agents are available as salvage therapies for refractory cases.

**Figure 2:**
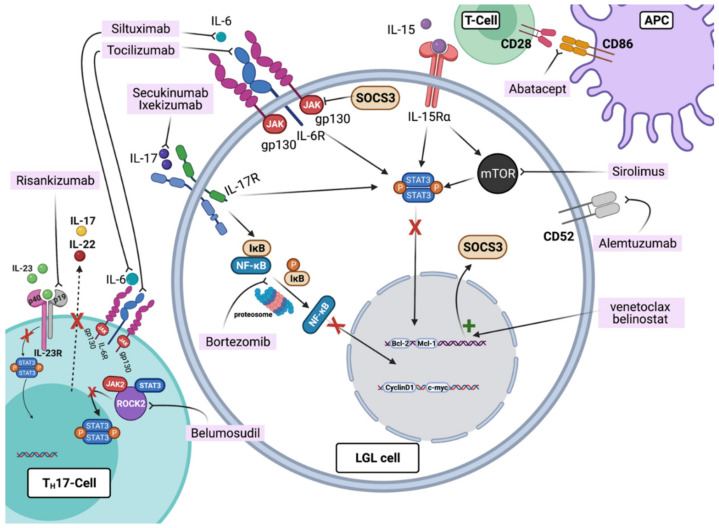
Pathways and agents described as having potential interest for the treatment of refractory LGLL. The humanized IgG1 Risankizumab is a mAB targeting the p19 subunit of IL-23 receptor and selectively inhibiting IL-23 and the STAT pathway, blocking IL-17 and IL-22 production. The ROCK2 inhibitor Belumosudil inhibits the STAT3 phosphorylation process. Anti-IL-6 mAB Tocilizumab and Siltuximab inhibit the JAK pathway by binding to the gp130 subunit. Secukinumab and Ixekizumab are anti-IL-17 mAB blocking the interaction with the IL-17 receptor with subsequent *STAT3* and *NF-kB* pathway inhibition. Bortezomib is a proteasome inhibitor that downregulates the *NF-kB* pathway activity, ultimately blocking the degradation of different pro-apoptotic factors. Abatacept, by binding to CD80/CD86 on APC, blocks the CD28-mediated APC and T-cell interaction. Sirolimus is an inhibitor of the mammalian-Target of rapamycin (*mTOR*) kinase pathway, leading to cytokine-mediated T-cell activation and *STAT3* inhibition. Alemtuzumab is anti-CD52, causing lymphocyte depletion through complement-medicated cell lysis and antibody-mediated cytotoxicity. Epigenetic modifiers such as Belinostat can restore *SOCS3* expression, which results in *STAT3* pathway inhibition. Figures were generated with BioRender.com (accessed on 20 August 2021).

Sirolimus (Rapamycin) interacts with the intracellular FK506 binding proteins (FKBPs) to form a complex that inhibits the mTOR kinase pathway, which is involved in physiologic cytokine-mediated T-cell activation and proliferation (Figure 2). Although results concerning LGLL patients treated with sirolimus are not well documented, our institutional experience and previous reports warrant its use in refractory cases, especially with associated anemia [14,44]. 

Alemtuzumab is a humanized anti-CD52 monoclonal antibody [45]. CD52 is ubiquitously expressed on all lymphocytes and attached to a glycosylphosphatidylinositol anchor, with a higher expression on CD4^+^ T-cells, moderate expression on CD8^+^ cells, and minimal/heterogeneous expression on NK-cells. Acquired resistance to alemtuzumab therapy in LGLL has been reported to be linked to CD52 down-modulation on LGLL cells [15,46], which eventually re-expresses CD52 upon discontinuation of the drug. ORR of around 50% has been consistently reported across different studies [17,43,45] (Table 1). Intravenous (IV) regimens modeled on therapy of B-cell non-Hodgkin lymphoma are associated with a significant risk of infectious complications, chiefly cytomegalovirus [47]. Modern subcutaneous (SC) regimens with a dosage of 10mg SC 1-2/week have completely replaced IV usage [48]. We recently reported the successful treatment of refractory T-cell LGLL patients with associated PRCA and *STAT3^Y640F^* mutation with this regimen [49].

Anti-thymocyte globulins (ATG) are polyclonal antibodies (IgG) targeting circulating T-cells via complement-dependent cell lysis and direct cytotoxicity leading to T-cell depletion. In addition, ATG has been shown to contain antibodies against several B-cell and NK-cell antigens [50], and is frequently used in hematopoietic stem cell transplant to prevent graft rejection and graft-versus-host disease, in the treatment of aplastic anemia and of the hypocellular form of myelodysplastic syndrome (MDS) [50,51]. Its use in LGLL patients has been limited to the refractory cases that failed 1st line therapy. With a previously reported ORR of 33% [43], we recommend using ATG in otherwise physically fit patients at a dose to be adjusted according to patient’s performance status with a treatment scheme of 40 mg/kg/day over four days duration borrowed from aplastic anemia and hypocellular MDS [43,52].

Rituximab is an anti-CD20 monoclonal antibody approved for the treatment of RA. A small retrospective study showed that treating LGLL patients with associated RA with this drug showed improvement in blood counts in patients with LGLL and RA with excellent tolerance and improvement of RA-specific symptoms. Such a response could be due to the suppression of IL-10, which subsequently suppresses STAT3 activation, explaining the particular activity in RA-associated LGLL cases which typically present with *STAT3* mutations. In addition, Rituximab may reduce T-cell activation and responsiveness by impairing antigen presentation processes due to B-cell depletion [53].

Bendamustine is a purine analog and an alkylating agent typically used in B-cell neoplasms. This agent also showed activity in patients with T-cell neoplasms, likely because of its pro-apoptotic effects. In particular, bendamustine has been anecdotally used to treat patients with LGLL with complete responses and improvement in blood counts after 3–6 months of treatment [54,55].

## 5. Potentially Available New Therapeutic Options and Clinical Evidence

The pathogenic overlap between autoimmune T-cell-mediated reactions and the reactive-to-semiautonomous nature of LGLL implies that many shared targets may exist in these conditions. Thus, refractory LGLL cases may be analyzed for the presence of clinical clues for rationally applied salvage therapies.

### 5.1. JAK-STAT Signaling Pathway Antagonists

Several compounds targeting JAK proteins have been proven to be effective therapeutic options in multiple diseases (e.g., Ruxolitinib in myeloproliferative disorders) [24] (Figure 2). Tofacitinib is a non-selective JAK inhibitor used for the treatment of RA, which primarily targets JAK3 but also has inhibitory effects on JAK1 and JAK2 [24,56]. This agent was found to be effective in patients with refractory LGLL and associated neutropenia by our group with a registered ORR of 67% in a cohort of nine cases. In vitro experiments confirmed an increased susceptibility of LGLL cells to the inhibitory effects of this medication, particularly when harboring *STAT3* mutations [57]. By comparison, Upadacitinib is a selective JAK1 inhibitor used in the treatment of refractory RA [58]. As a targeted JAK1 inhibitor, and given its success in RA, Upadacitinib may represent an interesting therapeutic option for the inhibition of the STAT3 pathway in refractory LGLL patients [24,59].

### 5.2. Inhibitors of T-Cell Activation

One hypothesis behind the persistent activation and proliferation of LGLL cells is the chronic exposure to “un-cleared” antigens [2]. For this purpose, medications inhibiting the cycle of T-cell activation, particularly in patients with associated autoimmune processes, provide a valid avenue for targeted treatment. Abatacept is a hybrid protein consisting of the extracellular domain of CTLA-4 linked to the Fc region of human IgG1 (CTLA4-Ig). Upon binding to CD80/CD86 on APC, abatacept blocks CD28 co-stimulation of naïve T-cells, attenuating their activation. Effective in RA [60], we previously reported the successful treatment of patients with refractory LGLL in association with RA and neutropenia with a response seen in 4/8 patients [43,57].

### 5.3. IL-6 Antagonists

IL-6 is a key cytokine activator of the JAK/STAT pathway [61]. In a homeostatic state, IL-6 binds to its membrane-bound receptor IL-6R or the soluble form sIL-6R, forming a complex with the corresponding transducer protein (gp130) [62]. This complex activates the JAK-STAT3 pathway and triggers the expression of the suppressor of the cytokine-signaling 3 (*SOCS3*) gene responsible for this physiologic negative-feedback found to be disrupted in LGLL [61,63]. Therefore, targeting IL-6 may represent a valid approach in refractory LGLL. Tocilizumab (anti-IL6 receptor) and Siltuximab (anti-IL6) are two approved drugs for the treatment of various autoimmune conditions (e.g., RA and Castleman disease) [62,64]. Considering the association of LGLL and RA [65], IL-6 antagonists may represent another therapeutic alternative, especially for LGLL patients with RA-like features. The associated risk of transient neutropenia with tocilizumab [65] warrants caution and suggests preferential use in refractory LGLL patients with transfusion-dependent anemia.

### 5.4. Multi-Cytokine Inhibitor BNZ-1

Multi-cytokine inhibitor BNZ-1 is a 19mer peptide that simulates gamma family cytokines (IL-2, -4, -7, -9, -15, and -21) and blocks specifically IL-2, -15, and -19 from interaction with the gamma receptor subunit CD132 without affecting other interleukins. Therefore, BNZ-1 is able to halt γ-chain receptor transduction signaling. The efficacy of this agent has been evaluated in ex vivo and in vitro studies, which showed that BNZ-1 led to apoptosis in LGLL cells with IL-2 and IL-15 proliferative responses [66]. Currently, a phase I-II trial is ongoing and is evaluating BNZ-1 in LGLL patients (NCT03239392). Preliminary data on its activity showed promising results, with a decline in T-cells and NK cells by 80–90% after two weeks from treatment start [67].

### 5.5. Proteasome Inhibitors

Bortezomib is a proteasome inhibitor that downregulates the NF-kβ pathway activity, ultimately blocking the degradation of different pro-apoptotic factors [68,69]. Given its efficacy in patients with multiple myeloma, this drug may be exploited in LGLL cases refractory to T-cell-directed immunosuppression, especially when associated with monoclonal gammopathies (Table 1). We previously reported the successful treatment with Bortezomib of patients with PRCA/LGLL with associated MGUS [70,71].

### 5.6. Epigenetic Modifiers

Epigenetic modulation is another mechanism involved in the pathogenesis of LGLL, as suggested by the disruption of the negative feedback of *SOCS3* during IL-6 mediated immune responses, the restoration of its expression in LGLL cells treated with 5-aza-2′-deoxycytidine (a hypomethylating agent), and the absence of mutations in this gene [61]. Of note is that histone deacetylase inhibitors are a group of epigenetic modulators that have been approved for the treatment of relapsed or refractory peripheral T-cell lymphoma (PTCL) [72]. Successful use of Belinostat and a modified low-dose non-cytotoxic regimen of decitabine in refractory LGLL have been previously reported [73,74].

## 6. Conclusions and Future Perspectives

Based on our current understanding of LGLL pathogenesis and the different cytokines involved in the perpetuation of the pathologic process, there is a compendium of targeted therapies approved for various autoimmune diseases that can be considered as potential new treatments for LGLL. For instance, Risankizumab is a humanized IgG1 mAB selectively inhibiting IL-23, producing complete inhibition of the IL-23 and IL-17 axis [75]. IL-23 has been found to induce IFN-γ production in T- and NK-cells, being able to activate the JAK-STAT pathway and ultimately resulting in the differentiation of TH17 cells [76,77,78]. These are specialized helper T-cells producing IL-17 and thereby influencing the NF-kβ pathway, and have been implicated in the pathogenesis of a variety of autoimmune diseases. IL-17 antagonists (e.g., Secukinumab and Ixekizumab) are commercially available and FDA approved for the treatment of ankylosing spondylitis and psoriatic arthritis, and may represent potential agents for LGLL, especially in the setting of association with autoimmune disorders [79,80,81,82,83] (Figure 2).

Another promising drug currently used for the treatment of graft-versus-host disease and potentially useful for the treatment of LGLL is Belumosudil, a Rho-associated coiled-coil-containing protein kinase-2 (ROCK2) inhibitor [84]. Having an essential role in coordinating TH17 cells, ROCK2 is known to be involved in T-cell mediated immune reactions [85]. Indeed, under perturbations of TH17 responses, ROCK2 isoenzyme aids in STAT3 phosphorylation with a subsequent intensification of immune dysregulation [85] (Figure 2).

LGLL is a fascinating disorder encompassing a variety of clinical presentations. Because of the rarity of the condition, randomized trials are difficult to conduct, and thereby management of refractory cases relies on empiric approaches, case reports, and series descriptions. A number of potentially useful agents, such as those mentioned above, are currently deployed to treat T-cell-mediated autoimmune diseases, of which some may also have utility in LGLL cases refractory to traditional agents.

## Figures and Tables

**Table 1:** Response to different therapies reported in the largest study cohorts of LGLL patients. The data presented are derived from a meta-analysis of the existing literature.

Treatments	Dong et al. 2021 [17]	Zhu et al. 2020 [14]	Bareau et al. 2010 [42]	Loughran et al. 2015 [40]	Sanikommu et al. 2018 [43]
*n*	319	108	229	59	204
Treated (%)	181 (57%)	105 (97%)	100 (44%)	55 (93%) 1st line (MTX) 14 (23%) 2nd line (Cy)	118 (58%)
MTX (n)	89	5	62	55	61
ORR	58 (56%)	0	34 (55%)	21 (38%)	26 (43%)
CR	14 (16%)	0	13 (21%)	3 (5%)	
Cy (n)	65	9	32	14	53
ORR	40 (62%)	7 (78%)	21 (66%)	9 (64%)	28 (53%)
CR	21 (32%)	5 (56%)	15 (47%)	3 (21%)	
CsA (n)	39	99 *	24	-	74
ORR	29 (74%)	49 (49%)	5 (21%)		36 (48%)
CR	9 (23%)	20 (20%)	1 (4%)		
Alemtuzumab (n)	6	-	-	-	24
ORR	3 (50%)				11 (46%)
CR	1 (17%)				

* 83 of 99 patients received CsA plus steroids.
